# Effects of the Interaction Between Genotype and Environment: Research into the Genetic Epidemiology of Alcohol Dependence

**Published:** 2002

**Authors:** Andrew C. Heath, Elliot C. Nelson

**Affiliations:** Andrew C. Heath, D.Phil., is Spencer T. Olin Professor of Psychiatry, and Elliot C. Nelson, M.D., is assistant professor of psychiatry. Both are associated with the Missouri Alcoholism Research Center and the Department of Psychiatry at Washington University School of Medicine, St. Louis, Missouri

**Keywords:** genetic theory of AODU (alcohol and other drug use), genotype, hereditary vs environmental factors, environmental factors, epidemiological indicators, AOD dependence potential, molecular genetics, AODR (alcohol and other drug related) genetic markers, aldehyde dehydrogenases, allele, comorbidity, behavioral and mental disorder, trauma, twin study, genetic correlation analysis

## Abstract

As researchers make progress in elucidating the roles of specific genes that contribute to the risk of alcohol dependence, they also need to understand how the interplay between genetic and environmental risk factors increases risk. The term “genotype × environment (GxE) interaction effect” refers both to the modification of genetic risk factors by environmental risk and protective factors and to the role of specific genetic risk factors in determining individual differences in vulnerability to environmental risk factors. Understanding the contributions of GxE interaction effects to the risk for and development of alcohol dependence and coexisting disorders is of paramount importance. These GxE interaction effects can be determined by appropriately designed family and molecular epidemiological studies, such as studies of children of twins and prospective family studies.

Research in genetic epidemiology concerns itself with “the role of genetic factors and their interaction with environmental factors in the occurrence of disease in human populations” (Khoury et al. 1993, p. 3). Two concerns motivate this area of study. First, standard epidemiological methods that ignore genetic effects on risk may reach erroneous conclusions in cases where genetic risk factors play a role in a disease. For example, using a purely epidemiological approach, the observation that alcoholism tends to run in families might lead to the inference that social learning processes are the main factor in its development. However, strong evidence indicates that the high correlation between first-degree relatives’ risk of alcohol dependence largely results from shared genetic risk factors rather than from purely environmental mechanisms (e.g., Heath 1995). Thus, adoption studies conducted since the 1970s have demonstrated that alcoholism risk in adoptees is correlated with the alcoholism histories of their biological parents rather than their adoptive parents (e.g., Goodwin et al. 1974, 1977; Cloninger et al. 1981).

Second, genetic studies that ignore the potential importance of environmental risk factors may have greater difficulty identifying genetic effects if such genetic effects are important (or especially important) only when the person is concurrently exposed to certain environmental risk factors (Khoury et al. 1993; Ottman 1990). For example, two genes involved in the metabolism of toxic substances associated with smoking—*CYP1A1* and *GSTT1*—predict the birth weight of the offspring only in mothers who smoked during pregnancy (Wang et al. 2002).

These observations suggest that it is the interplay of genetic and environmental risk factors that determines a person’s overall risk for alcohol dependence and other disorders. Researchers therefore may usefully consider the interactions between people’s genetic makeup (i.e., genotype) and their environments—the genotype by environment (GxE) interactions—when attempting to associate specific genes with the risk of specific disorders. This article first describes in more detail what GxE interactions are and discusses genotype–environment correlations. It then reviews the relationships between GxE interactions, alcoholism, and other coexisting psychiatric disorders. The article also presents an approach to investigating risk factors for alcohol dependence that allows researchers to determine GxE interaction effects while avoiding confounding variables. Finally, the article summarizes the implications of GxE interaction effects for the design of molecular epidemiology studies.

## What Is GxE Interaction?

The term “GxE interaction” refers to instances where the joint effects of genetic and environmental risk factors are significantly greater (or significantly reduced, in the case of protective factors) than would be predicted from the sum of the separate effects.^1^ Researchers who study genetic influences on behavior among members of a family (e.g., sibling pairs) have long distinguished between environmental influences that are shared by siblings reared in the same family and environmental influences that differ for those siblings (Jinks and Fulker 1970). Shared environmental (SE) influences could include parental influences that affect all siblings, effects of the neighborhood or school that apply to all siblings, shared peer influences, and similar factors. Nonshared environmental (NSE) influences or exposures, in contrast, are unique to each sibling, such as trauma experienced by only one child in the family. Except for studies of twin pairs who are reared in the same household and who are likely to be highly similar in their environmental experiences, however, this classification is clearly an oversimplification. For example, changes in the family’s socioeconomic status, moves to a new region or neighborhood, parental divorce and remarriage, and various other factors will have different effects on siblings who are raised in the same household but are of different ages.

The distinction between shared and nonshared environmental influences can provide useful insights into how GxE interactions contribute to the similarity in risk of alcohol dependence among family members. As suggested by Eaves and colleagues (1977), using “G” to denote genetic effects on risk,^2^ one can distinguish between genotype by shared environment interaction effects (GxSE) and genotype by non-shared environment interaction effects (GxNSE). The resemblance of various types of siblings reared together or apart then is determined by G, SE, NSE, GxSE, and GxNSE effects. The extent of the contribution of each of these factors depends on the type of siblings studied and their genetic relationships. For example, identical twins share 100 percent of their genes, fraternal twins and other full siblings share on average 50 percent of their genes, and unrelated people reared together (e.g., adopted and biological children, or step-siblings) share 0 percent of their genes. Table 1 summarizes the contributions of G, SE, NSE as well as GxSE and GxNSE interaction effects to the resemblance of siblings (also see Heath et al. 2002).

The contribution of GxE interaction effects to family resemblance for disorders such as alcohol dependence can be determined using some basic algebra. Assume that the total variation (V) in a population for a given index of alcoholism risk is the sum of all individual and interaction effects. This assumption is stated algebraically as V=G+SE+NSE+GxSE+GxNSE. The values of the various components in this sum can then be determined as follows (also see table 1):^3^

- Under relatively weak simplifying assumptions (e.g., that the parents are not genetically related), the contributions of genetic effects to sibling resemblance will be G for identical twins, 0.5 G for fraternal twins or full siblings, and 0 for genetically unrelated people. (In statistical terms, the “coefficients” for G will be 1.0 for identical twins, 0.5 for fraternal twins or full siblings, and 0 for unrelated people.)
- Nonshared environmental effects by definition will not contribute to sibling resemblance, only to the total variation in risk in the population. Thus, for all sibling relationships the coefficient for NSE will be 0.
- To simplify the analysis, shared environmental effects can be considered to make the same contribution to the resemblance of all types of siblings, regardless of their degree of genetic similarity, as long as they are reared together. Thus, for all sibling pairs reared together the coefficient for SE is 1.0. In practice, this is often an oversimplification because even common environmental factors may affect different people differently (e.g., because of their differences in age), particularly if they are unrelated.^4^ Similarly, one can assume that shared environmental effects make no contribution to the resemblance of twin and other sibling pairs reared apart (i.e., for sibling pairs reared apart the coefficient for SE is 0). Again, this assumption may be an oversimplification because it presumes that adoption agencies do not practice selective placement—that is, they do not place siblings in similar environments with respect to the religion, socioeconomic status, or other variables of the adoptive parents.
- Coefficients for multiplicative interaction terms (e.g., GxSE) in statistical analyses are derived by multiplying the coefficients for the individual variables (i.e., the coefficients for G and SE). Thus, interaction between genetic effects and shared environmental effects will contribute to the resemblance of genetically related people reared together but not to the resemblance of genetically unrelated people reared together or of genetically related people reared apart. Moreover, interaction between genetic effects and nonshared environmental effects will not contribute to sibling resemblance, but will contribute to the total variation in risk in the population.

The discussion so far has considered G as an abstract index of genetic risk; however, this approach to modeling GxE interaction effects can also be used to analyze the effects of specific genes^5^ on risk of alcohol dependence. Several investigators have discussed circumstances where an environmental risk factor may have an effect only in the presence of a particular variant of a gene (i.e., allele), or vice versa (e.g., Khoury et al. 1993; Ottman 1990). In practice, however, most risk factors likely contribute to risk even in the absence of another risk factor, although their joint effects might be greater (or less) than would be predicted if interaction effects are ignored.

How these statistical analyses apply to the effect of individual genes on risk can be illustrated by the following considerations. According to the basic rules of genetic inheritance each parent carries two alleles of a given gene, and each offspring inherits one copy from each parent. When one analyzes the alleles of that gene in full sibling pairs, in an average of 25 percent of pairs both siblings will have both inherited the same allele from their mother and the same allele from their father. That means that, with respect to that gene, the siblings will be genetically identical, like identical twins. In an additional 25 percent of sibling pairs, both siblings will have inherited different alleles from both parents and therefore will be genetically unrelated with respect to that gene. In the remaining 50 percent of sibling pairs, both siblings will have inherited the same allele from one parent and different alleles from the other parent, and therefore will share 50 percent of the genetic information for that gene (similar to the average prediction for full sibling or fraternal twin pairs in table 1). Thus, the simple algebra used above (and illustrated in table 1) is equally useful for understanding how individual genes interact with environmental risk factors to contribute to risk (Heath et al. 2002).

### The Role of Genotype–Environment Correlation

An additional issue that is relevant to understanding the inheritance of alcohol dependence risk concerns genotype– environment correlation (for an insightful early discussion, see Eaves et al. 1977). This term refers to the fact that people at high genetic risk for alcoholism may often be exposed to higher-risk environments as well. For example, they may be more likely to be exposed to heavy alcohol consumption by a parent in the home. Thus, if children with one or two alcohol-dependent parents have a higher chance of being in a high-risk environment for alcohol dependence or other psychopathology, then they may both inherit genetic risk factors and be more likely to be exposed to environmental risk factors. These two groups of risk factors may contribute to the children’s risk for alcohol dependence, both individually and through their interactions with each other. Such effects may also arise through the environmental influences of other biological relatives (e.g., older siblings) or, less obviously, through selective friendship or mate selection effects (Jacob et al. 2001). For example, impulsive people might have a tendency to select other impulsive people as peers or partners. Because evidence suggests that genetic factors influence impulsive personality traits (e.g., Martin et al. 1979), such a “selective mating” process can create a positive genotype–environment correlation if the impulsiveness of such peers or partners in turn has environmental effects on the person’s behavior (e.g., alcohol use).

## GxE Interaction Effects, Alcoholism, and Psychiatric Comorbidity

Several considerations strongly suggest that GxE interaction effects may be important in the development of alcohol dependence. For example, surveys that have assessed lifetime prevalence of various psychiatric disorders have noted that alcohol dependence and disorders such as depression, anxiety disorders, and history of childhood behavior problems (i.e., conduct disorder) occur together (i.e., are comorbid) much more frequently than would be expected by chance (Regier et al. 1990; Kessler et al. 1997*a*). Interpretations of such comorbidity have typically emphasized processes occurring within the person. For example, depression may be a risk factor for or a consequence of alcohol dependence; alternatively, both disorders may be influenced by common risk factors in the person.

It is also possible, however, that intergenerational effects (i.e., the transmission of genetic factors from one generation to the next), in interaction with the environmental influences of parents on their offspring, may be important in the development of psychopathology that is comorbid with alcohol dependence. The example of early childhood trauma (e.g., childhood sexual abuse) and its effects on later risk of psychopathology illustrate this possibility. A history of childhood sexual abuse is associated with an increased risk of alcohol dependence as well as an increased risk of a variety of other psychiatric disorders, including major depression, anxiety disorders, and conduct disorder (e.g., Kessler et al. 1997*b*). Results from twin studies suggest that this association is at least in part indirect, through other family background risk factors that are correlated with increased risk of abuse. These indirect pathways, which may involve intergenerational effects, are discussed in the following paragraphs. However, a direct association between childhood sexual abuse and subsequent psychopathology is also likely. For example, three studies evaluated twin pairs where one twin had been abused and the other had not. In all three studies, the abused twin was found to be more likely to report a history of psychiatric disorders when assessed as an adult than was the nonabused twin (Dinwiddie et al. 2000; Kendler et al. 2000; Nelson et al. 2002). These findings suggest that childhood sexual abuse can be a causal factor for the development of psychiatric disorders.^6^

These twin studies also provide information about whether indirect associations exist between early trauma, such as childhood sexual abuse, and risk of psychiatric disorder. For example, children who are abused are more likely to come from high-risk family backgrounds (e.g., a family with an alcoholic parent), and therefore are likely to be exposed to other environmental factors that may also predict increased risk of psychopathology. This interpretation is supported by the observation that in twin pairs where only one twin has been abused, the nonabused twin still is more likely to report a history of psychiatric disorder as an adult than are twins from families where neither twin has been abused (Nelson et al. 2002). Such an increased risk would be expected if additional familial environmental risk factors for psychiatric disorder existed that were more common in families where child abuse occurred.

One factor that is associated with a substantially increased risk of child sexual abuse, as well as with an increased risk of psychopathology, is having one or two alcohol-dependent parents (Dinwiddie et al. 2000; Nelson et al. 2002). Parental alcoholism also may be associated with a variety of other environmental risk factors, such as impaired parental supervision and parenting. Therefore, it is plausible that an indirect link exists that contributes to the association between parental alcohol dependence histories and the increased risk of psychopathology that is observed in the adult children of alcoholics (see the figure on page 197). Thus, parental alcohol dependence increases the risk of childhood abuse or other trauma for the children, which in turn is associated with an increased risk of later psychiatric disorder and may contribute to risk of alcohol dependence in the children.

However, people with the same history of trauma exposure (or exposure to other environmental risk factors) may differ in their risk of psychiatric disorder (e.g., depression) because of differences in genetic vulnerability (Sullivan et al. 2000). In other words, it is plausible that GxE interaction effects play an important role in determining risk. Because genetic factors contribute to the risk of alcohol dependence, offspring of alcohol-dependent parents will also inherit an increased genetic risk of alcohol dependence. Thus, in the presence of intergenerational processes such as those modeled in the figure, one would expect to observe increased rates of other psychiatric disorders in those with alcohol dependence, even if there were no direct effect of the comorbid disorders on risk of alcohol dependence. This is because alcohol-dependent people in addition to their genetic predisposition on average also are more likely to have experienced high-risk environmental exposures associated with parental alcoholism. These high-risk exposures, by interacting with genetic risk factors for other psychiatric disorders, will have increased the person’s risks of other psychopathology. In such instances where comorbid disorders do not directly influence the risk of alcohol dependence, the association between alcohol dependence and the comorbid psychiatric disorders (e.g., depression) would arise from intergenerational processes rather than from intrapersonal processes (also see the figure).

Although it is plausible that important GxE interaction effects exist, few studies have been able to document the importance of such effects in the development of alcohol dependence. In an early study using twin pair data, Heath and colleagues (1989) documented an apparent interaction between genetic effects on alcohol consumption level and marital status in Australian women. In that study, the genetic influence on current alcohol consumption levels was much stronger in women with no partner than in women with a current marital or de facto partner. Similarly, Dutch researchers have identified an apparent interaction between family religious background and genetic effects on impulsive personality traits that may be associated with increased risk of alcohol dependence. This study observed much stronger genetic effects in participants reared in a more permissive religious background (Boomsma et al. 1999). Finnish researchers have documented an interaction between place of residence (i.e., urban versus rural areas) and genetic effects on drinking behavior, with genetic influences being more important in urban settings (Dick et al. 2001).

**Figure:**
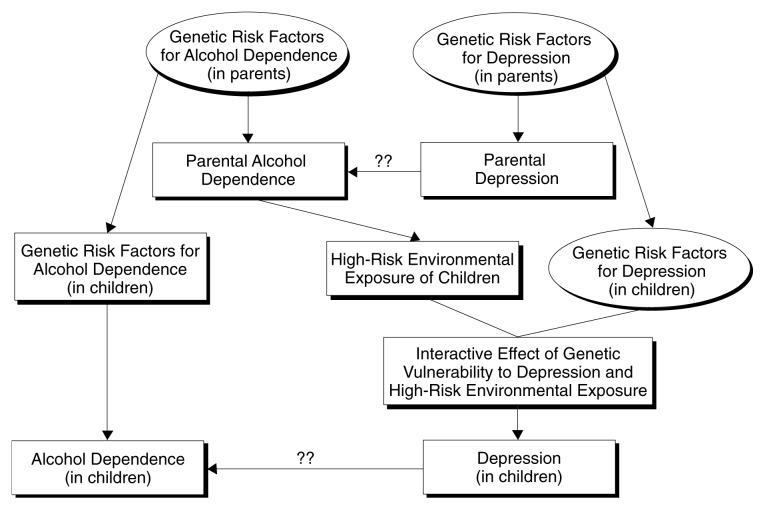
Schematic model showing how intergenerational processes, including genotype × environment interaction effects, may contribute to the development of alcohol dependence and comorbid psychiatric disorders, as illustrated by the example of depression. NOTE: ?? indicates uncertainty about whether depression directly affects the risk of alcohol dependence.

Perhaps the strongest evidence for GxE interaction effects in alcoholism etiology comes from Japanese researchers who analyzed the effects of the gene that carries the information for aldehyde dehydrogenase 2 (ALDH2), an enzyme that plays a pivotal role in alcohol metabolism in the body. Two alleles of the *ALDH2* gene exist. The *ALDH2–1* allele effectively converts acetaldehyde, a breakdown product of alcohol, into acetate and water. The *ALDH2–2* allele, however, is inactive, resulting in the accumulation of acetaldehyde, which exerts several toxic effects (e.g., a flushing response, nausea, and increased heart rate). Because each person inherits two copies of the *ALDH2* gene—one from the mother and one from the father—one can carry either two *ALDH2–1* alleles (i.e., be homozygous for *ALDH2–1*), one *ALDH2–1* and one *ALDH2–2* allele (i.e., be heterozygous), or two *ALDH2–2* alleles (i.e., be homozygous for *ALDH2–2*). People who are heterozygous experience a flushing response after consuming alcohol and have a substantially reduced risk of alcohol dependence compared with people who are homozygous for the functional *ALDH2–1* allele. People who are homozygous for the defective *ALDH2–2* allele experience an even more severe response to alcohol and have a risk of alcohol dependence that is close to zero. Thus, the *ALDH2–2* allele is said to have a protective effect on the risk of alcohol dependence.

The *ALDH2–2* allele occurs commonly in people of Asian ancestry but is not seen in most other racial groups. Higuchi and colleagues (1994) compared the frequencies of the *ALDH2–1* and *ALDH2–2* alleles in groups of Japanese alcoholics at different time periods. This analysis found that the protective effect of the heterozygous genotype was decreasing over time in Japanese males (i.e., the number of alcoholics who were heterozygous increased over time). The authors suggested that this pattern could be most plausibly interpreted as a GxE interaction effect, in which the increasing social pressures on Japanese males to drink with colleagues after work reduced the protective effect of the heterozygous genotype. Examples of such GxE interaction effects in the alcoholism literature are rare, however.

At first reading, much of the existing literature on the role of genetic factors in the development of alcohol dependence and other disorders (e.g., depression), particularly twin and adoption studies, appears to provide little support for the importance of family environmental and GxE interaction effects. These studies have consistently concluded that genetic factors overwhelmingly account for the familial transmission of risk of both alcohol dependence (e.g., Heath et al. 1997) and other psychiatric disorders, such as depression (e.g., Sullivan et al. 2000). Three issues, however, need to be considered before accepting this interpretation.

First, the frequency or severity of psychopathology in adoptive parents is likely to be lower than in biological parents who rear their own children. For example, in an adoption study conducted in Sweden, registration by the state for “alcoholism” was rare for adoptive parents compared with biological parents (Cloninger et al. 1985). In addition, adoptive parents typically are older than biological parents. Consequently, the range of high-risk environmental exposures of children reared in adoptive families is likely to be restricted.

Second, one must be careful in the interpretation of twin data. The ideal experiment for twin studies would be to compare identical and fraternal twin pairs reared together and apart. This design allows genetic effects, shared environmental effects, and GxSE interaction effects to be estimated jointly, as indicated by the coefficients summarized in table 1 (see Jinks and Fulker 1970; Eaves et al. 1977; Heath et al. 2002). However, separated twin pairs are extremely rare, particularly among younger cohorts. Therefore, twin studies of the etiology of alcoholism have used identical and fraternal twin pairs reared in the same household.

As shown in table 1, both in identical and in fraternal twins the coefficients for the genetic effects are identical to the coefficients for the GxSE interaction effects, indicating that both types of effects are confounded. In other words, the genetic variances estimated in twin studies will include the effects of GxSE interaction effects.^7^ One approach to separate the genetic and GxE interaction effects—the study of children of twins—is discussed in the next section.

The third consideration when interpreting twin studies is that of statistical power. In theory, it is possible to detect GxE interaction effects by stratifying relative pairs by high versus low environmental risk exposure. In practice, however, such analyses in most cases would require large sample sizes to reliably detect interaction effects. The sample sizes in many twin studies have not been large enough to allow detection of interaction effects.

### Studies of Children of Twins

Genetic research on alcohol dependence and other psychiatric disorders is still in the early stages of identifying genes that contribute to risk. Some important questions concerning GxE interaction effects can already be addressed in Asian populations, where the relatively high prevalence of the protective *ALDH2–2* allele allows for powerful analyses of interactions with environmental risk factors, as illustrated by the study of Higuchi and colleagues (1994). In populations of other ancestry, however, such analyses may need to await the identification of further genetic risk factors for alcohol dependence.

Researchers can, however, address the potential importance of GxE interaction effects using additional approaches to avoid the confounding with genetic effects that occurs in the traditional twin design. One such approach involves studying the children of twin pairs who are selected based on the presence or absence of alcohol dependence in the parent and his or her twin (see table 2) (Jacob et al. 2001). On average, the child of an alcohol-dependent twin may be expected to have a higher probability of exposure to a high-risk environment than the child of a nonal-coholic twin, particularly when other psychiatric disorders and psychopathology in the partners of the twins are controlled for. The child of an alcohol-dependent twin also will be on average at higher genetic risk, because genetic factors contribute to risk of alcohol dependence as well as to the risk of other disorders that may in turn contribute to risk of alcohol dependence. For children of nonalcoholic twins, the genetic risk for alcohol dependence depends on the alcohol dependence history and kind of genetic relationship of their parents’ co-twins. Thus, the genetic risk of children of a nonalcoholic twin will be highest if their parent has an identical twin who is alcohol dependent, intermediate if their parent has a fraternal twin who is alcohol dependent, and lowest if their parent has a fraternal or identical twin who is not alcohol dependent. Furthermore, GxSE interaction effects are expected to contribute to risk most strongly in children reared by an alcohol-dependent parent (that is, children who are at high genetic and high environmental risk), but to have reduced effects in children reared by a nondependent parent whose identical twin is alcohol dependent (i.e., children who are at high genetic risk but lower environmental risk).

Thus, in the children-of-twins design, genetic and GxSE interaction effects are not confounded in the same way that they are in the traditional twin study. It remains to be seen whether ongoing studies using this design will in fact support the importance of GxE interaction effects in the etiology of alcohol dependence and comorbid disorders.

## Conclusions and Implications for Molecular Epidemiology

As a result of the completion of the draft of the sequence of the entire human genome (Lander et al. 2001), investigators can anticipate rapid advances in ongoing efforts to identify genes that contribute to the risk of alcohol dependence. As progress in such efforts is made, it will become an urgent priority to conduct molecular epidemiological studies to investigate the effects of specific genetic risk factors as well as their interactions with other genetic and environmental risk factors and to determine how these interactions affect the risk of alcohol dependence in the general population.

The importance of such molecular epidemiological studies can be illustrated using the example of breast cancer. Even after researchers had identified a gene called *BRCA1*, which is associated with greatly increased risk of breast cancer, the implications of this gene for the breast cancer risk in the general population remained unknown for a long time because epidemiological studies were not carried out (Hopper 2001). Until such epidemiological studies were conducted, women in the general population (as well as women with a family history of breast cancer) could not be told how likely it was that if they underwent genetic testing, they would be found to have the high-risk gene.

When designing molecular epidemiological studies of the etiology of alcohol dependence, researchers can turn to commonly used epidemiological and genetic study approaches. Each of these approaches has some advantages and limitations, as described in the following paragraphs. In addition, study designs must consider the potential importance of GxE interaction effects and genotype– environment correlation effects.

Epidemiologists traditionally have used two study designs—case control studies and prospective cohort studies—that typically use samples of unrelated people (i.e., that do not include multiple family members in the same study) (e.g., Kleinbaum et al. 1982). In case control studies, investigators compare genetic or environmental risk factors for cases (e.g., alcoholics) and for unaffected control subjects (e.g., nonalcoholics). In prospective cohort studies, researchers follow a large cohort of initially unaffected people over an extended period of time to identify risk factors that predict subsequent disease onset. Geneticists, in contrast, typically use family studies and extended pedigree methods, often focusing on families in which multiple members are affected by a given disorder. In these studies, investigators analyze the co-occurrence among family members of a disorder and various genetic markers, attempting to identify markers that are inherited only by members who also develop the disorder. Case control studies using multiple genetic markers, however, which typically can have larger sample sizes and therefore greater statistical power, may soon replace traditional family studies (e.g., Risch 2000).

The primary advantage of case control studies is that they can use large samples (e.g., several thousand participants) and therefore have a high statistical power. Genetic case control studies also have some disadvantages, however, that may be avoided by family studies (e.g., Spielman et al. 1993; Spielman and Ewens 1998). For example, case control studies sometimes result in false positive associations between a marker and a disorder because of “population stratification”—differences in the frequencies of certain genes among groups of various ethnic or racial backgrounds. In family studies, where perfect matching for ancestral background is not an issue (e.g., because full siblings necessarily share the same ancestral background), this stratification is not an issue. The use of random genetic markers in a standard case control design may provide a cost-effective way of controlling for such confounding effects, however (e.g., Pritchard and Rosenberg 1999; Devlin et al. 2001). Because of their greater statistical power and the availability of techniques to overcome their disadvantages, case control designs are increasingly being used in molecular epidemiological research.

In the alcohol field, past experiences with case control studies of genetic association have been disappointing (e.g., Blum et al. 1990; Gelernter et al. 1993). It is possible, however, that with increased sample sizes and stringent statistical criteria for accepting a genetic association, such inconsistencies will disappear (e.g., Morton and Collins 1998). The validity of this approach has been demonstrated in the field of cardiovascular genetics, where studies contrasting as many as 5,000 cases and 5,000 control subjects have been completed (e.g., Cardon and Bell 2001). Such a sample size is adequate to analyze the effects of multiple genetic risk factors that individually have only modest effects on risk, as well as their interactions. Even such large case control studies have some limitations for establishing the effects of various environmental risk factors and their interactions with genetic factors. These limitations result from the retrospective design, which makes it difficult to reliably establish the participants’ past history of risk-factor exposure. To address this issue, large prospective cohort studies of adults, involving up to 500,000 participants, are being planned (Meade et al. 2002). These studies will document exposure to environmental risk factors and determine known and still-to-be-elucidated genetic risk factors for each participant. Thus, such studies have the potential to establish prospectively the effects of risk factors, genotype, and risk factor × genotype interactions on later disease outcomes.

If GxE interaction effects and similar intergenerational processes do indeed play an important role in the etiology of alcohol dependence, standard case control and prospective cohort studies using samples of unrelated people will have important limitations. These limitations become obvious when studying the model of GxE interaction effects shown in the figure. Under this model, given an adequately large sample size in a case control study, any gene that is a risk factor for alcohol dependence will also be identified as a genetic risk factor for major depression, even though the association with major depression arises solely via an environmental mechanism—the high-risk environmental exposures associated with the parent’s alcohol dependence that increase risk of depression in the parent’s offspring. In reality, however, such genes would influence the risk of depression only indirectly, through the environmental pathway from parental alcohol dependence to high-risk environmental exposures, which then interact with (other) genes that increase risk of depression (see Eaves and Sullivan 2001). Even controlling for alcohol dependence history in cases and control subjects will not remove this association, because it is the alcohol dependence histories of the parents and not of the cases or controls that contribute to the observed association. Furthermore, controlling for reported family history of alcoholism also is likely to be ineffective because such family history reports generally show low sensitivity (e.g., Rice et al. 1995).

An appropriate family study design can avoid such confounding effects (Eaves and Sullivan 2001). Equally important, studies involving multiple siblings from one family allow investigators to distinguish between direct effects of a risk factor and correlated effects of other risk factors related to the families’ background that may be more common in certain families. One example of such a situation is childhood sexual abuse, a childhood trauma with major effects on the risk of psychopathology. Thus, family studies can help determine if the risk of alcohol dependence is directly affected by childhood sexual abuse or by other family background factors that are more common in families where abuse occurs. In the former case, only the affected child would be at increased risk of alcohol dependence, whereas in the latter case all siblings, even those who had not been abused, would be at increased risk. Well-designed family studies can identify these differences, as well as the possible interactions of direct and indirect effects on risk with differences in genetic vulnerability.

For alcoholism, as for other disorders, one can best establish the importance of environmental risk factors and their interaction with genetic effects using prospective study designs. Psychiatric disorders, such as alcohol dependence, typically have an early onset, however (e.g., Nelson et al. 1996); therefore, prospective studies beginning in adulthood still would be largely retrospective with respect to risk factors for such disorders. For example, in the National Comorbidity Survey, a large psychiatric epidemiological survey, the median age-of-onset of alcohol dependence symptoms was around age 20. Important psychiatric predictors of increased risk of alcohol dependence, such as childhood conduct disorder, are of even earlier onset (Kessler et al. 1997*a*). Prospective studies of risk factors for alcohol dependence would therefore be initially pediatric studies. Such childhood studies have the advantage that they typically allow assessment of both the parent and the offspring generation as well as of multiple siblings from the same family, making this research both feasible and cost-effective.^8^ In the future, it seems likely that such prospective family studies will provide the basis for a better understanding of the genetic effects and GxE interaction effects on alcohol dependence and associated psychiatric disorders.

## Figures and Tables

**Table 1:** Contributions of Genetic and Environmental Effects, and Various Types of Genotype × Environment Interaction Effects to Sibling Resemblance and to the Total Variance in a Population

	Average Genetic Sharing	Genetic Effects (G)	Shared Environment Effects (SE)	Nonshared Environment Effects (NSE)	Genetic × Shared Environment Effects (GxSE)	Genetic × Nonshared Environment Effects (GxNSE)
Identical twins reared together	100%	1.0	1.0	0.0	1.0 × 1.0 = 1.0	1.0 × 0.0 = 0.0
Fraternal twins and full sibling pairs reared together	50%	0.5	1.0	0.0	0.5 × 1.0 = 0.5	0.5 × 0.0 = 0.0
Genetically unrelated siblings reared together	0%	0.0	1.0	0.0	0.0 × 1.0 = 0.0	0.0 × 0.0 = 0.0
Identical twins reared apart	100%	1.0	0.0	0.0	1.0 × 0.0 = 0.0	1.0 × 0.0 = 0.0
Fraternal twins and full sibling pairs reared apart	50%	0.5	0.0	0.0	0.5 × 0.0 = 0.0	0.5 × 0.0 = 0.0
Total population variance	—	1.0	1.0	1.0	1.0 × 1.0 = 1.0	1.0 × 1.0 = 1.0

NOTE: A coefficient of 1.0 indicates a high contribution, a coefficient of 0.5 an intermediate contribution, and a coefficient of 0 no contribution. SOURCE: Heath et al. 2002.

**Table 2:** Use of the Children-of-Twins Design to Separate Genetic and Environmental Influences on Risk of Psychopathology in the Children of Alcohol-Dependent Parents

		Level of Risk to Children Due to:		
		
Parent's History of Alcohol Dependence	Alcohol Dependence History of the Parent's Twin	Genetic Effects	Familial Environmental Effects^a^	Genotype × Environment Interaction Effects
Alcohol dependent	Any	High	High	High
Nondependent	Alcohol dependent, identical twin	High	Low	Low
Nondependent	Alcohol dependent, fraternal twin	Intermediate	Low	Low
Nondependent	Nondependent	Low	Low	Very Low

a including other shared environmental effects associated with parental alcohol dependence.

NOTE: Predictions assume statistical control for psychopathology in the partners of the twins (i.e., the biological co-parents of their children), and for psychiatric comorbidity in the twin parents. SOURCE: Jacob et al. 2001.

## Footnotes

1 As in other areas of statistics, it is traditional in genetic statistics to model the interaction of genetic and environmental effects as a multiplicative term, hence the “x” in “GxE.”

2 The term “genes” in this article specifically means those genes for which different variants exist in a given population and that can therefore contribute to observed differences in risk in the population.

3 For a good introduction to such analyses from a statistical perspective, see Sham 1997.

4 An exception to this statement may be pairs of unrelated people of the same age who are reared in the same family.

5 The same applies to regions on the chromosomes that are believed to contain genetic risk factors but for which researchers have not yet identified specific genes.

6 An important limitation of these findings is that all three studies were retrospective—that is, the adult twins reported whether they had ever been abused. Thus, it is possible that recall or reporting biases could have contributed to this association, although it seems implausible that such biases could completely explain this association.

7 This issue applies equally when considering the contributions of specific genes to the familial transmission of alcohol dependence.

8 At the same time, research on pediatric populations involves some additional logistical complications, such as obtaining parental consent and appropriate child assents to participation in research that includes both diagnostic assessments and genetic analyses, as well as mandated reporting requirements for serious events such as sexual abuse of a child.
